# Cancer Prevention and Therapy by Targeting Oxidative Stress Pathways

**DOI:** 10.3390/molecules28114293

**Published:** 2023-05-24

**Authors:** Sarmistha Saha, Luciano Saso, Guliz Armagan

**Affiliations:** 1 Department of Biotechnology, Institute of Applied Sciences & Humanities, GLA University, Mathura 281406, India; 2 Department of Physiology and Pharmacology “Vittorio Erspamer”, Sapienza University of Rome, 00185 Rome, Italy; luciano.saso@uniroma1.it; 3 Department of Biochemistry, Faculty of Pharmacy, Ege University, Bornova, Izmir 35100, Turkey; guliz.armagan@ege.edu.tr

## 1. Introduction

Oxidative stress arises from the inadequate production of reactive oxygen species (ROS) which couldn’t be neutralized by antioxidant defense. Cells maintain this balance of oxidants and antioxidants by different biochemical, metabolic and genetic mechanisms and in case of imbalance, several pathophysiological consequences can occur [1]. The cross-talk between cancer and oxidative stress is contradictory, either promoting tumorigenesis and cancer cell proliferation or stimulating apoptosis. The proliferation of cancer cells is accompanied by ROS overproduction; however, tumor cells balance this ROS threshold via an array of antioxidant systems to avoid ferroptosis, apoptosis or senescence [2]. On one hand, ROS scavenging by antioxidants could increase apoptosis and thus, deprives cancer cells of energy in early tumorigenesis. On the other hand, ROS production could selectively kill tumor cells by apoptosis, autophagy, necrosis and ferroptosis [3]. Therefore, the fine tuning between the ROS production and clearance is highly crucial. Among the key antioxidant systems, glutathione and thioredoxin pathways and Nrf2/Keap1 signaling systems have been reviewed in cancer [3]. In this context, peroxidation of the polyunsaturated fatty acids in the lipid bilayer of the cell membranes also gives rise to 4-hydroxynonenal (4-HNE), also known as the second messenger of ROS, which has been shown to exhibit a crucial role in cancer [4]. 4-HNE reportedly promotes cell proliferation, however, in some conditions it can stimulate apoptosis or necrosis of specific cancer cells [5]. 4-HNE exerts its effects by either binding to the arginine, histidine and cysteine residues of proteins, or with DNA leading to their modifications [6,7].

It is assumed that some non-cancer cells also associate with tumors, such as cancer-associated fibroblasts (CAFs), which cooperate to maintain the tumor homeostasis and stimulate the proliferation, progression and invasion of tumor cells. CAFs and ROS have a bilateral relationship, ROS, particularly H_2_O_2_, attack fibroblasts and convert them into active CAFs by upregulation of the expression of HIF1α [8]. On the other hand, an increase in ROS-generated CAFs promotes cancer growth and invasiveness, which in turn leads to the upregulation in the antioxidant gene expressions [9]. Accumulating evidence suggests that the oncogenes raise ROS production. Thus, RAS and STAT3 genes activates NOX2 and NOX4 gene by altering mitochondrial membrane potential [10,11]. Similarly, BCL-2 and MYC are also responsible for ROS production by altering mitochondrial function [12,13]. Also, the down-regulation of tumor suppressor mediated gene expressions of antioxidant genes such as *SOD2*, *GPX1*, *SESN1* and *SESN2*, also promote the generation of ROS in tumor cells [14]. Moreover, tumor cells are also stimulated by the TNFα released from the immune cells [15]. For instance, breast cancer cells activate Ca^2+^ signaling via redox sensitive TRPA1 channels, which in turn activates PI3K-PKB/Akt pathways and oxidative stress tolerance [16]. On the other hand, *TRPA1* gene expression is also controlled by Nrf2 pathway, suggesting the possible link between Ca^2+^ signaling and oxidative stress [16].

Nrf2 (NF-E2-related factor 2) is a transcription factor, which belongs to the group of Cap’n’collar (CNC) family of bZIP transcription factors [17]. Nrf2 along with its negative regulator, the E3 ligase adaptor Kelch-like ECH-associated protein 1 (Keap1), cooperate to regulate the intracellular redox homeostasis in cancer and other diseases [17]. In normal circumstances, Keap1 binds with Neh2 domain of Nrf2 via ETGE and DLG motifs, leading to the localization of Nrf2 in cytoplasm [18]. However, with oxidative stress, Nrf2 doesn’t interact with ubiquitin-conjugating system due to a conformational change in the E3-ligase complex [18]. This results the Nrf2 release from the complex, leading to the translocation of the Nrf2 into the nucleus, heterodimer formation with sMaf protein and ultimately leads to the ARE activation, which further regulates cell protective mechanisms and antioxidant proteins expressions [19]. Nrf2 pathway promotes the expression of genes including TXN, G6PD, GSTA2, NQO1, and HMOX1 which are related to NADH regeneration and redox detoxification [20]. This special issue shares insights on the therapeutic strategies by targeting redox signaling pathways in cancer. The study by González-Montero et al. [21] documents the role of ascorbate as a potent therapeutic agent in cancer. The authors discuss the paradoxical effects of ascorbate, which induces oxidative stress at high concentration by interacting with iron. Also, it was suggested that ascorbate could also act as an adjuvant in different cancer therapies by inducing apoptosis and ferroptosis. 

We consider this Special issue to bring together a wide range of review and research articles contributing to an understanding of the cancer therapies based on redox state.
